# Recombinant Acidic Fibroblast Growth Factor Facilitates Motor Recovery and Reduces Myelomalacia in Traumatic American Spinal Injury Association Impairment Scale A Spinal Cord Injured Patients

**DOI:** 10.1089/neur.2024.0063

**Published:** 2024-10-02

**Authors:** Wan-Ya Chang, Wen-Cheng Huang, Yun-An Tsai, Lin-Hsue Yang, Yi-Tien Su, Shih-Fong Huang, Chiau-Li Huang, Ya-Hui Lee, Shu-Shong Hsu, Li-Yu Fay

**Affiliations:** ^1^ Eusol Biotech Co., Ltd., Taipei, Taiwan.; ^2^ Institute of Pharmacology, National Yang Ming Chiao Tung University, Taipei, Taiwan.; ^3^ School of Medicine, National Yang Ming Chiao Tung University, Taipei, Taiwan.; ^4^ Department of Neurosurgery, Neurological Institute, Taipei Veterans General Hospital, Taipei, Taiwan.; ^5^ Division of Neurosurgery, Department of Surgery, Far Eastern Memorial Hospital, New Taipei City, Taiwan.; ^6^ Department of Rehabilitation, Far Eastern Memorial Hospital, New Taipei City, Taiwan.; ^7^ Division of Neurosurgery, Department of Surgery, Kaohsiung Veterans General Hospital, Kaohsiung, Taiwan.

**Keywords:** aFGF, motor score, MRI, myelomalacia, spinal cord injury

## Abstract

This study aims to evaluate the potential benefits of treating spinal cord injury (SCI) patients with acidic fibroblast growth factor (aFGF), a potent neurotrophic factor that preserves neuronal survival. The study involved 12 tetraplegic patients with American Spinal Injury Association Impairment Scale (AIS) Grade A SCI who were randomly assigned to receive either a recombinant human aFGF or a placebo every 4 weeks for three doses. Participants underwent comprehensive evaluations of medical, neurological, and functional parameters at baseline and every 4 weeks after the first dose until the 48th week. The first dose was administered directly to the injury site during surgery within 6 weeks of the SCI, while the subsequent two doses were administered via lumbar puncture with a 4-week interval. The results revealed promising beneficial effects of aFGF on AIS Grade A SCI patients. The study report highlights aFGF’s potential to expedite motor recovery in complete SCI patients and significantly increase the probability of a 10-point improvement when compared to the placebo group (odds ratio = 6.06, *p* = 0.0004). Furthermore, aFGF treatment exhibited a significant reduction (*p* < 0.01) in the incidence or exacerbation rate of myelomalacia, a known secondary complication following SCIs.

## Introduction

Traumatic spinal cord injury (SCI) is a neurological condition that can cause motor, sensory, and autonomic deficits. It significantly impacts the daily lives of patients and their families. There is currently no approved pharmacological intervention for traumatic SCI. It is reported that acidic fibroblast growth factor (aFGF) facilitates neuron regeneration by downregulation of proteins that inhibit neuronal regeneration. It helps spinal cord repair after injury by induction of neuroprotective protein factors involved in the spinal cord repair process.^1–3^ Several single-arm, open-label clinical trials observed that aFGF was safe and improved function in chronic SCI patients.^4–6^ Our study aims to evaluate the potential benefits of treating SCI patients with aFGF. The data of this study were from a multicenter, double-blind, randomized clinical trial registered at clinicaltrials.gov (identifier NCT 03229031) to evaluate the efficacy and safety of aFGF in acute spinal cord injured patients. The trial was terminated prematurely due to slow patient recruitment during the COVID-19 pandemic. However, their data remain valuable in exploring the potential of aFGF’s clinical benefit because it is derived from a double-blind, placebo-controlled, randomized study. The results may provide insight into better ways to treat SCI. We explored whether aFGF treatment facilitates motor recovery and exerts diminution in secondary changes after SCI. Relevant information from this trial was therefore used for this purpose.

## Materials and Methods

As mentioned above, the data of this study were from a prior clinical trial, which is the first multicenter randomized clinical trial (RCT) for aFGF in SCI. It screened 20 SCI subjects; 15 were eligible and randomized, including 12 American Spinal Injury Association Impairment Scale Grade A (AIS A) and 3 AIS Grade B (AIS B) SCI patients. They were stratified by the study site and randomly assigned to receive either aFGF (20 µg) or a placebo every 4 weeks for three doses. The RCT was approved by institutional review boards of three medical centers in Taiwan. All participants gave written informed consent before enrollment in adherence to ethical principles and local laws and regulations. This report only analyzed the data of the 12 AIS A subjects to minimize the heterogeneity of a small sample size.

The AIS grade was determined based on the International Standards for Neurological Classification of Spinal Cord Injury (ISNCSCI, REV 11/15). The AIS A was defined as complete injury having no sensory response to light touch and pinprick, no response to deep anal pressure at the sacral segments S4-5, and no voluntary anal contraction preserved. ISNCSCI ASIA motor scores were measured as the sum of upper and lower extremity motor scores by trained rehabilitation physicians before the treatment at the laminectomy and neurolysis surgery and every 4 weeks until 48 weeks after the first treatment dose. All assessments were conducted without knowledge of previous assessment results to avoid bias.

According to the ICCP panel guidelines^7,8^ and observations of Waters et al.^9^ and Marino et al.,^10^ a patient with low-cervical (C4–C7) and complete SCI is likely to spontaneously improve ∼4.6–10 points motor score after the first year of injury. Therefore, the “10-point increase” in motor score is set as a response threshold for analysis. The response time indicated by a 10-point improvement in ISNCSCI motor scores is compared between the responders in aFGF and placebo groups.

A whole spine MRI scan was performed to evaluate spinal cord lesions and monitor the secondary changes that affect the spinal cord following traumatic injury. All patients underwent three times of MRI without sedation with the following pulse sequences: sagittal SE T1WI, sagittal FSE proton weighted and T2 weighted images (PDWI and T2WI), sagittal STIR, and axial SE T2WI at the baseline, 24 weeks, and 48 weeks after the first dose of aFGF or placebo treatment, respectively. MRI results were used to analyze the SCI-related secondary changes in the spinal cord before and after aFGF treatment.

All patients’ vital signs, adverse events, medication discontinuation due to adverse events, physical exams, clinical safety laboratory data, ECGs, and gallium scans were monitored or conducted for safety every 4 weeks throughout the trial period. Quality of life, psychological disorders, and suicidal ideation were also monitored regularly.

The aFGF used in the RCT is an investigational recombinant human aFGF. It is produced through *Escherichia coli* and is composed of 135 amino acids with a molecular weight of ∼15.2 KDa. The recombinant aFGF was manufactured and provided by Eusol Biotech Co., Ltd., a Taiwan FDA-certified GMP facility.

The study used SAS statistical software (version 9.4) for analysis. Descriptive statistics were applied, and continuous variables were summarized with means and standard deviations or standard errors. As emphasized in the SCI trial guideline,^7^ the ASIA motor score changes may not follow a normal, bell-shaped curve because of the underlying physiology and the natural history of spontaneous recovery. This may make normal-theory statistical procedures like the *t*-test and analysis of variance inapplicable in small samples.

Analysis of changes in motor score using geometric mean can be less affected by extreme values in a skewed distribution. Motor scores were analyzed using a repeated measure mixed model with a random intercept for each subject, accommodating missing data, assuming that the missing data is missing at random. Mixed models for repeated measures (MMRM) analyses provide a comprehensive way to analyze longitudinal continuous end-points of clinical trials to avoid model misspecification and with its unbiasedness for data missing completely at random. The model included changes in log-transformed motor scores from baseline to the targeted time as response variables. The fixed effects included treatment, visit, treatment-by-visit interaction, and injury level and motor score at baseline as covariates. An unstructured covariance matrix was used for measurements within the same subject, assuming that measures for different subjects are independent. Least-squares mean differences and the associated 95% confidence intervals (95% CIs) were provided using MMRM. A logistic regression analysis was performed to evaluate the likelihood of achieving a 10-point increment in motor score. The difference in occurrence/worsening of myelomalacia between the two groups monitored by MRI scan was examined using a chi-square test.

## Results

Among the 12 tetraplegic complete SCI patients, five patients with cervical SCI at the C4–C6 motor levels were treated with three doses of recombinant aFGF (20 µg). Their mean age at enrollment was 38.8 ± 15.1 (min–max:19–53) years, and the mean injury duration before the first treatment dose was 30.8 ± 7.3 (min–max:20–38) days. The mean baseline motor score was 8.8 ± 6.0 (min–max:0.0–17.0). Seven patients in the placebo group had the same injured motor levels; their mean age was 26.0 ± 13.6 (17–56) years, and the mean injury duration before the first treatment dose was 30.9 ± 6.0 (23.0–42.0) days. Their mean baseline motor score was 14.4 ± 8.1 (9.0–29.0). The two groups had no statistical difference in the motor scores, ages, and injury durations at the baseline.

**FIG. 1.**
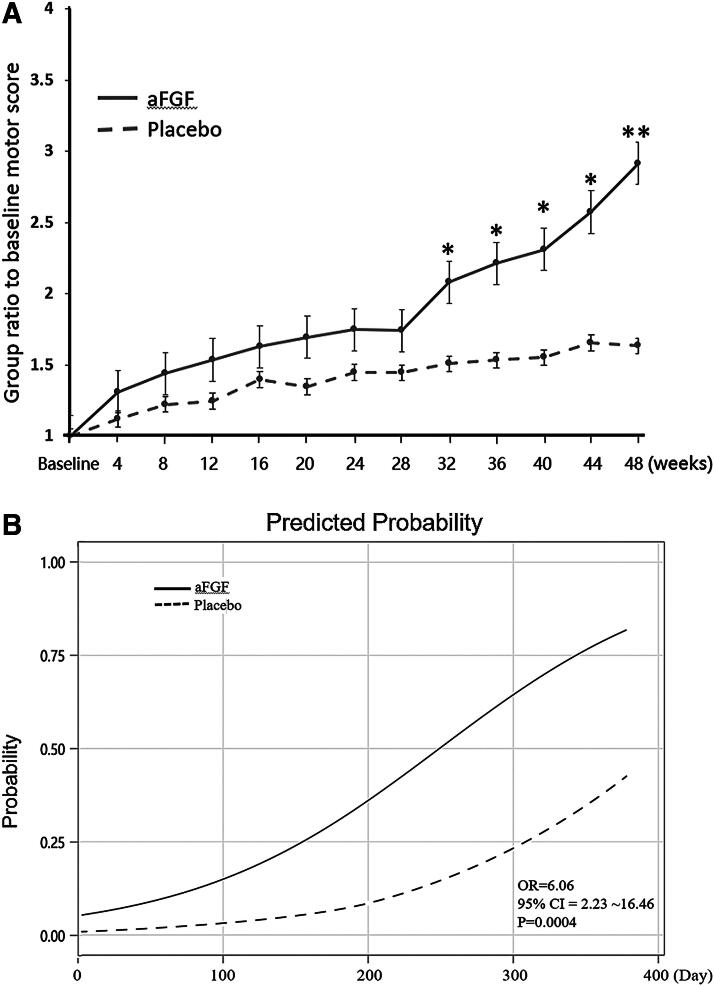
The changes in group ratio to baseline geometric mean of motor score **(A)** and the predicted probability of 10-point motor score improvement in aFGF and placebo groups **(B)** over time after treatment. The ratio is calculated as the geometric mean of the treatment average divided by the geometric mean at baseline (*p < 0.05; **p < 0.01). aFGF, acidic fibroblast growth factor; CI, confidence interval; OR, odds ratio.

The aFGF and placebo groups showed no significant difference in all safety data. No drug-related adverse event/severe adverse event was observed in either group. Patients who received aFGF showed a larger increment in group ratio to baseline motor score than the placebo group (Fig. 1). Statistically significant differences in the increment between the aFGF and placebo groups were observed from the 32nd (0.0408) to 48th week (0.0064) after the first treatment dose (Fig. 1a). While the rates of improvement in motor scores achieved a 10-point were similar in both groups (2/5 = 40% vs. 3/7 = 43%), there was a statistically significant difference in response time to achieve the 10-point motor recovery. For the two patients treated with aFGF, it took them 57 and 138 days (mean ± standard deviation [SD], 97.5 ± 57.3 with 95% CI of 18.1–176.9) to improve 10 points in their motor scores. On the contrary, for the three patients who received the placebo, the 10-point motor score improvement took them 223, 259, and 280 days (mean ± SD, 254 ± 28.8 with 95% CI of 221.4–286.6), respectively. The odds of achieving a 10-point motor score increase was significantly higher in the aFGF group than that in the placebo group (Fig. 1b). The odds ratio was 6.06 with a 95% CI of 2.23–16.46 (*p =* 0.0004).

The MRI reports showed that only one out of the seven (1/7) patients in the placebo group had myelomalacia at the baseline and whose lesion had extended from C6/C7 to C4/C7 at the 24th week. Myelomalacia occurred in five more patients of the placebo group and made up a total of six out of seven (6/7) patients based on the MRI evaluations at the 24th and 48th weeks (Table 1). As for the five patients who received aFGF, the baseline MRI reports showed that two out of five (2/5) patients already had myelomalacia, but no exacerbation of their lesions was observed by MRI at the 24th and 48th weeks. There was no additional case that occurred in the aFGF group in the follow-up evaluations at the 24th and 48th weeks. Chi-square analysis indicates that the placebo group had a significantly higher rate (6/7) of secondary injury (including worsening) compared to the aFGF group (0/5) based on the initial and follow-up whole spine MRI scans. The calculated value of *X*^2^ from the chi-square analysis is 8.571 (*p* < 0.01). After Yates’ correction, the difference remains statistically significant, with an *X*^2^ value of 5.486 (*p* = 0.0152).

**Table 1.** MRI Reports Showing the Change in Patient Numbers (Rate) of Developing Progressive Myelomalacia After Traumatic SCI in the aFGF and Placebo Groups Before and at the 24th and 48th Weeks After Treatment

	MRI result	Treatment		Total
Time	Myelomalacia	ES135 (*n* = 5)	Placebo (*n* = 7)	(*n* = 12)
	*n* (Missing)	5 (0)	7 (0)	12 (0)
Baseline	Yes	2 (40.0%)	1 (14.3%)	3 (25.0%)
	No	3 (60.0%)	6 (85.7%)	9 (75.0%)
Week 24	Yes	2 (40.0%)	6 (85.7%)	8 (66.7%)
	No	3 (60.0%)	1 (14.3%)	4 (33.3%)
Change from baseline	Worsening	0	6 (85.7%)	6 (50.0%)
	No change	5 (100%)	1 (14.3%)	6 (50.0%)
Week 48	Yes	2 (40.0%)	6 (85.7%)	8 (66.7%)
	No	3 (60.0%)	1 (14.3%)	4 (33.3%)
Change from baseline	Worsening	0	6 (85.7%)	6 (50.0%)
	No change	5 (100%)	1 (14.3%)	6 (50.0%)

For the between-group difference in post-treatment occurrence or exacerbation of myelomalacia, the chi-square is 8.571, *p* < 0.01. The difference remains statistically significant after adjusted by the Yates’ correction, X^2^ = 5.486, *p* value is 0.0152.

aFGF, acidic fibroblast growth factor; SCI, spinal cord injury.

## Discussion

Despite the limited patient number, it was a well-controlled study with randomization to protect blindness and avoid potential bias of outcome measures. The MRI reports of the placebo group were consistent with the observation reported by Planner and colleagues,^11^,^12^ in which they observed a focal small region of myelomalacia at and approximately one vertebral segment cephalad to the injury site present on the MRI in all AIS A SCI patients (*n* = 10) on recovery (between 3 and 14 months). Our study findings may be the first demonstration that a pharmacological treatment can reduce the incidence of subacute progressive myelopathy and prevent the exacerbation of existing myelopathy in patients with severe SCI. It is worthwhile to study further if aFGF may prevent subacute progressive myelopathy after SCI and its clinical significance.

Accurately assessing SCI outcomes or benefits of therapeutic interventions is complex and challenging. In real-world clinical practice, “Time is Spine” means that SCI should be treated earlier rather than later.^13^ However, acute spinal shock may happen within 72 hours and last to 4 weeks or longer after SCI.^7,14^ To minimize the impact of acute spinal shock on baseline assessment, the mean injury duration of 12 enrolled patients who received the first dose of aFGF or placebo was 30.8 ± 6.2 days.

The complexity of motor function recovery that makes prediction difficult is another limitation. For instance, the same magnitude of changes in motor score may come from very different baseline conditions, such as from 0 progressing to 5 or from 13 to 18. These changes may represent completely different clinical significance. On the contrary, quite different outcomes may be seen in patients with the same baseline motor scores or AIS grading due to different zones of partial preservation, which may affect the spontaneous recovery or degree of recovery achieved later. All these diverse situations multiply the difficulties in predicting recovery outcomes in SCI clinical investigation. The improvement in ASIA motor recovery has been well accepted as a reliable efficacy endpoint for predicting functional outcomes after SCI. However, the effectiveness of a pharmacological approach in preventing secondary myelopathy that hampers the recovery of complete SCI patients may also be considered as an important indicator to predict motor recovery in future studies.

## Conclusions

This is the first report providing clinical evidence that aFGF significantly reduces myelomalacia, one of the major secondary injuries after SCI, and potentially aids motor recovery in complete SCI patients. Despite the limited sample size and exploratory nature, the results make heuristic sense and demonstrate the beneficial effect of aFGF on SCI, which may help future study designs to improve the relevance of SCI research. It is worth exploring the relationship between myelomalacia and motor recovery and necessary to investigate further to confirm the findings and to develop an optimal treatment regimen with confirmatory clinical trials to prevent subacute progressive myelopathy and facilitate recovery in SCI patients.

## Abbreviations Used

ECGs: Electrocardiograms
FDA: Food and Drug Administration
FSE: Fast spin echo
GMP: Good manufacturing Practice
ICCP: International Campaign for Cures of SCI Paralysis
MRI: Magnetic Resonance Imaging
PDWI: Proton Density Weighted Image
STIR: Short tau inversion recovery
